# Moral and Physical Massage

**Published:** 1887-04-09

**Authors:** Stretch Dowse


### Moral and Physical Massage.

By Dr. Stretch Dowse.

A Lecture delivered at the Midwives' Institute and Trained Nurses' Club, Buckingham Street.

I.—Explanatory and Descriptive.

We divide massage into two distinct classes, namely, Moral and Physical. By the former I mean massage not only by the hand, but by the spirit, by the will, and by the intellect. By the latter we mean massage by the hand pure and simple, in fact, mechanical massage or medical rubbing. All who have had experience in manipulating the body by the hands and by the fingers, must have been occasionally struck with wonderment when they have felt the response to the influence they were exerting upon their patients. I know of nothing more fascinating than this form of moral influence, which I admit is frequently the outcome of a peculiar individuality which can neither be taught nor acquired. This intuitive moral faculty or individuality of impression can only be engendered by the special influence which a strong mind invariably has upon a weak one. Remember it is not the brute form of mind which possesses this faculty, not the mere mind of courage and daring which resists everything and is at the same time devoid of reciprocity; it is rather the mind of sympathy with decision, the mind which is associated with natural affection, the mind full of purpose guided by confidence and success, full of hope and disdainful of failure,—such a mind, in fact, which rallies a beaten army, endows the soldier with courage, crushes dread and fear, and leads on to death or victory; such a mind can so influence fellow-minds as to raise up a storm of rage and indignation, or a feeling of sympathy akin to love, or a sense of admiration, fascination, bewilderment, amazement, and stupefaction. It is a power which is capable of cultivation; it is a power which appeals to the senses rather than to reason; it is a power, in fact, by which influence and control are exerted and made evident and manifest. Although I feel that I am treading upon dangerous ground, still I cannot leave this subject, which appears to me of some importance in discussing the meaning of moral massage, without drawing your attention to the special influence it exercises over moral or mental pain. In all matters relating to pleasure or to pain on the moral or the emotional side of human nature, I am unable to draw a definite line of demarcation between such conditions and the pain which is associated in a part injured or disabled and diseased. The more I become acquainted with the varying phases of aberration of reason, and moral and mental change, the more am I convinced that every mental process—that is, every process of ideational transformation has its analogue in certain physical transformations—not of the brain alone, but of the sensory side of the body generally; and if this be so, we can the more readily understand why a mind blunted to ordinary moral impressions is invariably associated with a body whose physical sensibility is in like manner impaired.

We can instance this a little further. A mind diseased with exalted, perverted, and exaggerated ideas, delusions, or even hallucinations, is invariably associated with a body the sensibility of which is altered and perverted. The man who is deluded with the idea that he is the Prince of Wales, is not unlikely to put his hand into cold water and say it is hot, and to his perverted sensibility it feels hot, although to ordinary senses it is cold. I bring forward these few simple instances to show you how the mind and body work hand in hand, how the mind can influence the body, and how the body must influence the mind, and how essential the health of the one is to the health of the other. Unity is always strength; disunion means disintegration, disease, and weakness.

I think from these few remarks, and in a lecture of this kind I cannot go more deeply into this subject, you will perceive what I mean by Moral Massage. And now for a word or two about mental pain as influenced by moral massage. The most common instances of imaginary pain are those which are called hysterical. Mental pain may be considered as a consciousness of that which disturbs the harmony of the mental factors. I need scarcely tell you that there are a thousand ills in life by which this unfortunate consummation can be realised: the chief of these are jealousy, love, religion, and avarice. Physical pain is so well known to everyone that I need scarcely give you an instance, —perhaps toothache is the most common and most widely diffused of all physical pain. Let me tell you that the physiology of pain is exceedingly mysterious. Physiology cannot yet define with exactitude why the racking pain of a decayed tooth departs like magic at the sight of the cold steel, and perhaps does not return for months, if it ever does. The fearful pains associated with locomotor ataxy are exceedingly peculiar, and demonstrate perhaps more clearly than any other form of pain how the moral and the physical are connected and correlated. We find that even the worst form of pain will yield to the soothing influence of moral massage. Moral massage is both inductive and seductive, it works inductively by an act or process of reasoning from a part to a whole, or from particulars to generals; it works seductively, inasmuch as it leads the mind of the sufferer away from the region of imaginary pain. I will make my meaning understood. Supposing I were going to massage a painful spot which is not uncommon under the left breast. I should begin by effluraging the part with the tips of my fingers, and the patient would in all probability declare she could not bear it. I should then turn my attention to petrissaging or pinching the similar spot on the opposite side of the body, until I had produced in it a decidedly painful impression. Continuing to massage this spot, I should keep my patient's attention fixed upon it, and I should then transfer my massage to the original painful spot; and by so doing I should, by careful manipulation, have full play over the part which was the original seat of pain.

We next have to consider the individuality of the massuer and the masseuse, and we will divide these into the physical and the moral, but we can, after what I have said of moral massage, consider them both together. Some individuals are utterly unfitted for this office, both by nature, by education, and by general development. I have no wish to lay down a hard and fast line as to physical development, to the entire exclusion of a large number of persons who fail to come up to the exact standard, for it must be remembered, and it is a point to be borne in mind, that our patients to be massaged are not all of the same standard either mentally, morally, or physically. Young children, for instance, do not require the standard that would be required for a fully developed adult. The standard of individuality for the masseur or the masseuse may be thus defined :—

1. Good physique and good health absolutely essential.
2. Cleanliness in every particular is of the greatest importance.
3. An intelligent interest in the patient's welfare.
4. Perfect devotion and zeal in carrying out fully and carefully the duties of the work, so as to ensure the confidence of the patient.
5. Absence of fuss and undue haste.
6. Good temper and forbearance are necessary.
7. Intelligence, and even refinement, are advantageous.
8. A happy, cheerful disposition, with vivacity and brightness, dexterity, readiness, and ability, not forgetting a pleasant contented face, completes the standard of individuality. But now comes the important part of all, and that is, practice in the art. I need scarcely tell you that it is of no use your having all the qualifications necessary, for without practice you are useless. It is only practice in manipulations, guided by some knowledge of anatomy and physiology, which can make perfection in the art of massage.

I now consider the massage hand,—and it is a point of such great importance that I am constrained to speak of it separately. The natural position of the hand is certainly in a measure indicative of the character of the individual; for instance, the hand of the Venus de Medici is what may be termed the sensitive nervous hand,—there is the slightly drooping wrist, the extension of the metacarpophalangeal joints, slight bending of the fingers with the thumb drawn backwards; whilst in the Diana of the British Museum, which is the typification of a strong woman, the hand is free, the wrist extended, and the fingers and thumb flexed. This condition of hand is usually considered to be indicative of will, determination, energy, and power; and it is well known that persons in whom the thumb is largely developed, have energy, will, and individuality. These are passing points of interest, and are worth remembering. The perfect hand for massage work should be soft, smooth, dry, and fleshy, and of good normal healthy temperature. The square hand with powerful thumb adductors is usually found most useful, but the following points in the nature of the hand are of unquestionable value: muscular power, suppleness, pliability, flexibility, firmness of grip, and compliancy to yield readily, impressibility, smoothness, fineness, warmth, even delicacy, and freedom from moisture, are essential. You will find that every part of the hand must be made available, and there is no position which the hand and fingers can assume, as we shall see presently, which cannot be adapted to certain forms of massage.

Whilst considering the masseur, I am anxious to draw your attention to several points of the greatest importance. The first is, that you keep yourselves in good health, take three good meals a day, but never take stimulant until your work is over, then, provided you are in active work, you will find a bottle of stout and a good night's rest restore your energy, and equip you for the following day's work. Avoid working on Sundays, if you possibly can, and on this day get fresh air and natural exercise. Now clearly understand me upon this point. If you are in active work you will have to conserve your energy in every way possible. You cannot, for instance, walk a mile, even to your patient, for, should you get to your patient flushed, hot, and wearied, you are doing yourself and your patient an injustice. And now, if you please, we will consider our patient from several points of view. The room should be of comfortable temperature, say from 62 deg. to 65 deg. F. The couch or bed upon which the patient is placed should not be too soft or yielding. As little as possible of the body of the patient should be exposed at one time. There is never any necessity to expose the abdomen, but the extremities and the back and loins can be worked better when uncovered.

In general massage, one hour should lapse after a meal before the process is commenced, and the process should extend from thirty to forty minutes, twice a day, between eleven and twelve in the morning, and between five and six in the evening, or between twelve and one mid-day, and eight and nine o'clock at night. If a joint alone is to be massaged, then the process need not extend over fifteen or twenty minutes, but in cases of general paralysis and other forms of muscular wasting, the process should never be less than half an hour, and it should be repeated three times a day, namely, at eleven a.m., four p.m., and seven p.m. Some writers on massage state that the duration of the *séance* should not last more than eight or ten minutes. This is all very well for an individual joint, but to say that the body should be slurred over in ten minutes simply shows lamentable ignorance.

After the operation is over, it is imperative that the patient should be made thoroughly warm, and be kept at rest for half an hour. I want you, if you please, to remember this question of warmth after every form of massage, whether local or general: "Always endeavour to maintain the heat which your energy has generated and created." When it is necessary for you to perform petrissage, and pinch and knead the deeper muscles, where you want to exercise energy and pressure, the skin can be advantageously washed with a spirit and ammonia lotion. What I commonly use has the following composition :—℞, Liq. Ammon. fort. ℥ss., Aq. Aurantii flor. ℥j, Eau de Cologne ℥j, Spirit Vini Rect. ad ℥viij. Vaseline and other tar compounds are highly objectionable, and so are preparations bearing the name of electron.

(*To be continued.*)

